# Exploring the Role of Provider–Patient Communication in Women’s Sexual Health and Pre-Exposure Prophylaxis Care in the Primary Care Settings in New York State of the United States

**DOI:** 10.3390/ijerph19138084

**Published:** 2022-07-01

**Authors:** Chen Zhang, Kevin Fiscella, Yu Liu

**Affiliations:** 1 School of Nursing, University of Rochester, Rochester, NY 14627, USA; 2 School of Dentistry and Medicine, University of Rochester, Rochester, NY 14627, USA; kevin_fiscella@urmc.rochester.edu (K.F.); yu_liu@urmc.rochester.edu (Y.L.)

**Keywords:** patient–provider communication, interpersonal relationship, PrEP care implementation, sexual wellness, sexual health

## Abstract

Background: Women shoulder a disproportionate burden of HIV infection in the United States and worldwide. Pre-exposure prophylaxis (PrEP) is an effective tool for HIV prevention, but its use is suboptimal. Primary care providers (PCP) are considered the ideal PrEP caregivers, but they generally underperform in PrEP care implementation. Methods: From 2020 to 2022, we employed semi-structured in-depth interviews to collect information about barriers and facilitators in PrEP care and beliefs and opinions regarding sexual wellness among 18 PCP and 29 PrEP-eligible women. We employed content analysis and thematic analysis to explore the transcribed narrative data. Results: The current study was guided by the “Communication Pathways” Framework. We studied how communication functions affect PrEP care and women’s sexual wellness on the pathways. We identified several specific pathways between communication and PrEP care implementation in primary care settings, including patient knowledge, linkage to care, therapeutic alliance, and decision making on PrEP care. A paradox regarding who should initiate the discussion regarding sexual history and PrEP care was identified. Conclusions: Findings suggested that a navigation and assistance system for PrEP care in patients and providers is urgently needed. Future studies should facilitate PrEP discussion, engagement, and monitoring in primary care settings.

## 1. Introduction

Globally, it is estimated that nearly 20 million women and girls are living with HIV, accounting for 53% of the HIV-infected population [1]. In 2019, approximately 1.2 million people were living with HIV in the United States (U.S.), with about 35,000 new infections [2]. To curb the HIV epidemic and broaden HIV prevention strategies, the U.S. Food and Drug Administration approved daily oral pre-exposure prophylaxis (PrEP; brand name Truvada^®^ (Foster City, CA, USA) (emtricitabine/tenofovir disoproxil fumarate) and Descovy^®^ (emtricitabine/tenofovir alafenamide)) for primary HIV prevention for individuals at elevated HIV risk based on sufficient evidence from multiple clinical trials [3,4,5,6,7,8,9,10]. Clinical trials have demonstrated the efficacy of HIV PrEP when taken with high adherence among high-risk populations [3,4,5,6,7,8]. Several studies indicated that, with sustained optimal compliance, PrEP could reduce the risk of infection by more than 90% [11], suggesting that PrEP can be an effective biomedical HIV prevention method. 

Although health professionals have made tremendous efforts, the uptake and provision of PrEP among people at risk of HIV acquisition has been slow [12]. Nationwide, there is a significant disparity between the number of people who have indications for PrEP use and those who use PrEP for HIV prevention [13,14]. Women who bear disproportionate burdens in the HIV epidemic are much less representative in the current PrEP user profiles than their male counterparts [15]. Specifically, women made up only 6% of current PrEP users, while they accounted for 18% of new HIV infections in 2020 [15]. The primary transmission route for a woman contracting HIV is via heterosexual contacts [16], and women usually have the least control over safe sex practices in these scenarios [17,18]. In particular, social inequalities combined with limited women-controlled HIV prevention strategies urgently need a tool that can empower women to mitigate the heavy HIV burden among them [17,19]. 

PrEP is efficacious, safe, cost-effective, and particularly suited for women [20]. PrEP trials in women have demonstrated the importance of medication adherence for PrEP effectiveness, making implementation of PrEP among high-risk women feasible and desirable [21,22,23]. Specifically, PrEP showed high biological efficacy under optimal adherence [24,25], with about 95% (95% CI, 0.70 to 0.99) protection among subjects with a detectable drug level [24]. Furthermore, PrEP use among women has several features [26]. Unlike in males, PrEP can be administered either orally (e.g., pills) or vaginally (e.g., gel), with flexible frequencies (e.g., daily vs. on-demand) [27]. Thus, the provision of PrEP for women at risk of HIV acquisition has become a top priority and a significant research gap. 

To facilitate PrEP provision, Nunn and colleagues proposed a “PrEP implementation cascade” model, which suggests that progression along stages of the cascade must involve interaction and engagement among patients and health providers in the system [28,29]. Primary care providers (PCP) are considered the ideal PrEP providers as they usually encounter HIV-negative patients with indications for PrEP use. They can offer excellent continuity of care to achieve optimal effectiveness [30]. However, PCP generally underperform in PrEP care implementation compared to peers specializing in HIV care or sexually transmitted disease (STD) treatment [29]. For instance, in a recent meta-analysis, Zhang et al. (2019) reported that the odds of the willingness to provide PrEP care and the actual PrEP prescription among infectious disease specialists were three (odds ratio (OR) = 3.06, 95% CI = 2.27, 4.11) and four times (OR = 3.98, 95% CI = 3.11, 5.10) the odds among PCP, respectively [29]. Previous scholarly work identified two key barriers that may hamper the PrEP implementation, including “lack of PrEP request from patients” [29] and “lack of screening for patients’ risky behaviors” [31]. Both barriers reflect the disconnection in interpersonal communication between health providers and patients, “the most important but least accomplished” component in health care [32,33]. 

Research exploring the association between patient–provider communication and health outcomes reported mixed results from favorable to null [34]. The discrepancies may be due to different study designs (e.g., randomized control trials vs. cross-sectional study design), measurements (e.g., operational vs. conceptual), or broader contextual factors (e.g., side effects, transportation) [32,33,35,36]. Furthermore, the “Communication Pathways” framework suggests that communication functions (e.g., information exchange) may, directly and indirectly, affect health-related proximal outcomes (e.g., provider–patient agreement, trust) and intermediate outcomes (e.g., linkage to care, PrEP care) [33]. These communication functions may impact optimal health outcomes through multiple pathways (e.g., improved access to care, enhanced patient knowledge and shared understanding, enhanced therapeutic appliance and patient empowerment, and better quality decisions) [33]. As one of the pioneering works [30,37], we aimed to explore how specific communication pathways and interpersonal relationships between health providers and their patients impacted the PrEP care implementation in the primary care settings in this current study. Only a few studies have explored opinions regarding PrEP care from both sides nor assessed communication functions specifically [30,37]. Therefore, our analysis explored the unique role of communication in PrEP care in the primary care settings between providers and patients with the guidance of a theoretical framework. 

## 2. Materials and Methods

### 2.1. Participants’ Recruitment and Screening

Potential participants, including PCP and women with PrEP indications, were recruited from September 2021 to March 2022 from multiple routes (e.g., ResearchMarch^®^ portal, newsletters, clinic flyers, and social media). The research team conducted a screening survey combining demographics and eligibility criteria either by phone or by email to assess if the potential participant was eligible for this study. Eligible women were those who (1) were 18 years or older, (2) resided in N.Y. state, (3) had at least one visit with a PCP in the past 12 months, (4) had PrEP indications based upon the CDC guidance [38,39], (5) were English speaking, and (6) were willing to provide consent. Eligible health providers need to (1) be 18 years or older, (2) primarily practice in N.Y. state, (3) practice as a primary care provider and have prescription privileges, (4) be English speaking, and (5) be willing to provide consent. With the distributed recruitment messages, interested participants contacted the research team for further screening. 

### 2.2. Sample Size, Data Collection and Data Analysis 

**Figure 1:**
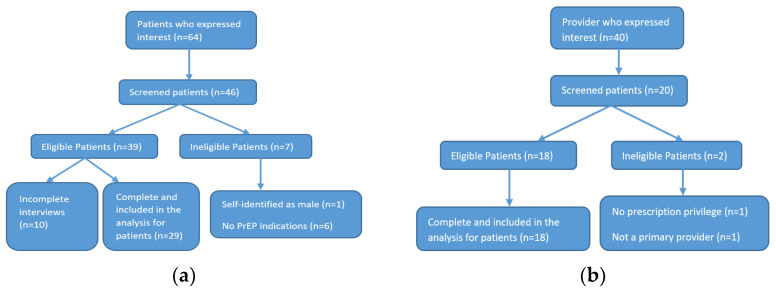
Flowchart for participants enrollment: (**a**) Flowchart for patients enrollment; (**b**) flowchart for providers enrollment.

Once study subjects passed eligibility screening and provided the consent form, semi-structured interviews were conducted by ZOOM or by phone, based upon the individual’s preference. We used either a Health Insurance Portability and Accountability Act (HIPAA)-compliant digital recorder or HIPAA-compliant ZOOM with participants’ consent to record interviews. Overall, a total of 64 patients were contacted by the research team, 46 proceeded to eligibility screening, and 29 completed the interview. For providers, 40 contacted the research team, 20 were screened, and 18 were eligible and completed the interview (Figure 1a,b). 

**Figure 2:**
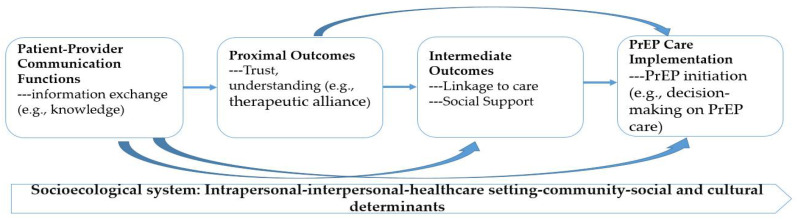
Integrated model of Communication Pathways on PrEP care implementation.

According to the empirical evidence concerning the sample size sufficiency for semi-structure interviews [40,41,42], we recruited enough participants to ensure the “information power” could be reached (i.e., semi-structured/in-depth interviews require a minimum sample size of between 5 and 25) [43]. A 30 min semi-structured in-depth interview was conducted with each participant. During the interview, we used the pre-established interview guideline to probe and elicit responses from our participants regarding several key components (e.g., communication functions, proximal and intermediate outcomes on PrEP implementation cascade) based upon the Communication Pathways Framework [33]. We identified several specific pathways between communication and PrEP care implementation in primary care settings, including patient knowledge (i.e., communication function), therapeutic alliance (i.e., proximal outcomes), linkage to care (i.e., intermediate outcomes), and decision making on PrEP care (PrEP implementation) (Figure 2). 

These semi-structured interviews elicited information regarding how specific communication pathways affected the PrEP care continuum among PrEP-eligible women and primary care providers in the primary care settings. For PCP, we ascertained opinions that may facilitate and impede PrEP provision, patient interaction, decision making in PrEP initiation and care delivery, and potential solutions that may ease the communication procedure (i.e., conveying and receiving messages). For PrEP-eligible women, we explored their experience of doctor visits, interactions with PCP, and possible solutions that may facilitate their communication with their health providers regarding PrEP screening and PrEP consultation experience.

Before analyses, all interview tapes were transcribed verbatim (except for identifying information). All qualitative data were de-identified. We employed content analysis and thematic analysis to analyze the transcribed narrative data [44,45]. We used an iterative approach to developing and refining a coding dictionary to enable data retrieval and analysis throughout the data collection procedure [46]. In the first phase, three investigators familiarized themselves with the transcribed data by independently reading a few transcripts at the initial stage of codebook development. To facilitate codebook development, we extracted theoretical constructs from existing literature. We coded texts using coding themes related to the central questions in our interview guides and new themes that emerged in the coding process. Then, each investigator independently read, coded, and categorized the qualitative transcripts to ensure inter-researcher reliability and resolve discrepancies. In the final phase, all investigators identified themes and patterns based on the codebook. Content areas were characterized by their emergent themes and some quantification of these via the content-coding results. This process was continued until no new themes emerged. Data were primarily analyzed based on the identified theoretical framework. We used a qualitative software (i.e., ^®^Atlas.ti) to facilitate the data analyses. 

## 3. Results

### 3.1. Participants’ Characteristics 

We included 18 providers and 29 PrEP-eligible women in the data analysis in the current study. For providers, the average age was 42.4 years old (standard deviation (SD) = 12.9, range 28 to 68). The majority practiced in urban settings, with one in a rural area and three in suburban areas. Among them, 13 were female, and 17 were White, with only 1 self-reported Black. For women, their mean age was 38.1 (SD = 15.1, range 20–61). Among these women, only five reported ever taking PrEP, and the rest were PrEP naïve. Almost half of the included women reported multiple partnerships in the past six months (Table 1). 

### 3.2. Key Findings Based upon the Framework 

Using the framework, we identified several specific pathways between communication and PrEP care implementation in primary care settings, including patient knowledge (i.e., communication function), therapeutic alliance (i.e., proximal outcomes), linkage to care (i.e., intermediate outcomes), and decision making on PrEP care (PrEP implementation). We also situated these specific pathways within the broader context of intrapersonal (e.g., demographics), interpersonal (e.g., provider–patient relationships), healthcare settings (e.g., patient navigators and assistance), community (e.g., community support), and societal determinants (e.g., norms and stigma) that may be associated with health outcomes and health utilization. We further identified a “discussion paradox” phenomenon regarding the initiation of the PrEP care and sexual wellness in primary care settings. We related these findings with the “Communication Pathway” Framework to collectively interplay how specific communication pathways and interpersonal relationships between health providers and their patients impacted PrEP care implementation in the primary care settings (Table 2). 

#### 3.2.1. The Status of PrEP Care Implementation in the Primary Care Settings 

We thoroughly explored how provider–patient communication can affect PrEP care implementation status in the primary care settings through several pathways, including patient knowledge (e.g., perceived HIV risks), linkage to care (access to care, navigating assistance), therapeutic alliance (e.g., foresting relationship; supports from health professionals), and decision-making procedure (trust in health providers and system; shared understanding) [47,48,49]. 

##### Pathway via Patient Knowledge (i.e., PrEP Awareness and Perceived Risks) and Linkage to Care 

Studies showed that PrEP awareness and linkage to care were critical entry points into the PrEP cascade and served as the foundation for achieving protection from HIV with PrEP [50]. To start the cascade, patients must have an accurate understanding of the risks and benefits of PrEP use to make an informed decision with the assistance of their health providers. Health providers also need to clearly understand patients’ needs, values, preferences, and beliefs about their health [51]. Communication serves as a bridge to facilitate the understanding of each other’s values, preferences, and points of view [52]. 

In the current studies, we found that the majority of the interviewed women (>90%) perceived themselves as having a low risk of HIV infection, despite frequently practicing high-risk sexual behaviors (e.g., unprotected sex, having multiple concurrent partners). For instance, a woman who had condomless sex with multiple sexual partners rated herself as “low to moderate” risk, as she explained: “*I’d say (I have) a low to moderate risk (of HIV infection). I-I don’t use, um, condoms, but—and I-I do have multiple partners, but, um, they, based on kind of, like, the lifestyle, um, that I know that they-that they’re in, um, I believe that they’re also low risk, so, um, I don’t—and I try to—and I-I never really, like, specifically asked, but, um, I just assumed, based on the type of people that I, um, interact with*.” (women#19, 20 years). 

Another woman shared a similar rationale about how to rate HIV risk for herself when she had unprotected casual sex, and she reported that she solely relied on her intuition to make a judgment on her sexual partner’s risk.

“*I would say um, I had a little less than moderate (risk of HIV infection), not super light…but, um, I’m—I don’t use drugs, and I’m not using it to contact those people who use drugs. I tend to have, um, other casual, sexual partners, I do not use condoms, and I just assume that the other person is safe, even though I don’t truly have everything condom using. I just assume that the person is safe enough for me to not use it. But it’s not based on any proof, or it’s not like I check specific for HIV negative. Yeah. It’s just a judgement on my part. I don’t know if their HIV—status is negative.*”

(women#22, 22 years)

To engage patients in PrEP care, a risk assessment of their PrEP eligibility is an essential gateway. However, patients’ false impressions about their HIV risks may hinder providers’ ability to further explore patients’ PrEP candidacy; as one provider illustrated, *“I’ve been trying to get it more with women but with only limited success. I’ve had limited success in getting people to—to accept it…I-I just haven’t—I haven’t got a lot of buy-in… definitely a lower perceived risk. accepting the medication is kind of like a-admitting to themselves, like, I can’t trust my boyfriend. I’ve had patients who’ve had a lot of trouble with pills. They’ll just say, ‘I don’t like pills in general.’ Or they find it to be large and—and uncomfortable and difficult to swallow.”* (PCP#26, 48 years, female, primary care).

Furthermore, the dialogs about PrEP use often require open discussions about sexual activities or other risky behaviors (e.g., unprotected sex, multiple partners). Some health providers made ungrounded assumptions about their patients’ risk profiles to avoid the discomfort. As a provider indicated *“I don’t use any kind of standardized (PrEP eligibility screening) tool in my practice. Many of patients are either, um, not in any kind of a relationship at all, or, uh, in stable relationships that they’re not outside of the relationship.”* (PCP#16; 54 years, female, family medicine). 

In addition to the limited PrEP knowledge and reluctance to communicate sexual behaviors in healthcare settings, linkage to care was constrained by a lack of navigation assistance in the complex health system, especially for PrEP care that requests relatively intensive resources. About one-third of the interviewed providers mentioned the navigator would be great assistance to PrEP care in their practice. A PCP illustrated how the presence of a navigator could impact PrEP care implementation in healthcare settings, using an example by comparing the difference between her previous and current practice clinics.

“*In my old practice, we had PrEP navigators that would actually kind of help on the back end of getting, um, you know, the PrEP app filled out, and any sort of, um, drug assistance programs that the patients might qualify for. But, that’s just not a resource that I have in my current practice. a lot of times if I am running into cost barriers, um, I do, um, end up just referring patients to a local practice that I know is much more capable of navigating that. it’s just a lot easier when you can kind of have all of that information right at the time of the visit.*”

(PCP#14, 38 years, female, family medicine)

Although limited knowledge and suboptimal linkage to care may hinder PrEP care implementation, pathways via increasing patient knowledge and strengthening linkage to care could be achieved by clear and open communication between providers and patients. For instance, a PCP described her experience of smoothly introducing PrEP to her patients who had misconceptions about their own HIV risks.

“*I think there is a misconception that only men who have sex with men should consider being on PrEP…it’s important when I talk to patients about it that, um, patients recognize that anyone is at risk for HIV who has any sexual activity. I pretty much talk about safe sex practices with all my patients regularly, not just at their health visits, but at many other visits as well… I don’t think I get immediate negative reactions from patients when I bring it up… I try to normalize the reaction by saying, you know, a lot of the messaging regarding PrEP has been to certain groups of people who we think might be more at risk for contracting HIV, but we know the risk of contracting HIV lies in anyone who has unprotected sex, which is why I bring it up with all my patients who are having unprotected sex.*”

(PCP#38, 29 years, female, family medicine)

##### Pathway through Establishing the Therapeutic Alliance and Facilitating Decision Making on PrEP Care 

The therapeutic alliance refers to an alliance that involves a mutually trusted relationship between patients, health professionals, and significant others (e.g., friends, families). A trustworthy alliance facilitates optimal health treatment outcomes [53]. The alliance can be enhanced when health providers express their understanding and empathy towards patients while patients themselves can express concerns in a non-judgmental environment [53]. In the current study, we found that PCP were fostering an alliance with patients in PrEP care by being positive and supportive about PrEP use for patients with PrEP indications, and they believed that PrEP could protect individuals who engaged in risky behaviors, as one of the providers endorsed:

“*PrEP is a fantastic harm reduction strategy. Um, and as a HIV provider, um, I find it incredibly useful for, um, patients who, um, potentially have partners, uh, who are sero-discordant, um, or in sero-discordant relationships to protect the partner. people who maybe, um, engaged in a little bit riskier behavior. Um, so that we can, uh, keep them healthy. Yes, there are some possible risks with it, but overall, in a young, healthy individual, um, the benefits of it often significantly outweigh the risks.*”

(PCP#14, 38 years, female, family medicine)

Furthermore, from patients’ perspectives, the therapeutic alliance in primary care settings facilitates them to become familiar with and accept PrEP use for their HIV prevention. A patient thoroughly described her experience from being suspicious to fully acceptable PrEP with the alliance formed:

“*…he (my PCP) was the one that discussed (PrEP)… At that time, he (my HIV-positive husband) wasn’t doin’ too well, so he (my PCP) was like, ‘Well, he doesn’t like usin’ condoms. You know, you have to use protection.’ He (my PCP) told me about Truvada. And it upset my stomach a little bit when I first, ‘, started usin’ it, you know, but then, after a while, I felt better about takin’ it…No, it wasn’t embarrassing, no…but he (my PCP) was like, you know, ‘You need to take care of yourself. You know, you need to take care of him too. If you’re not using condoms, this is the best thing I could give you, but, also, use condoms.’ and he (my PCP) gave me a pamphlet and stuff like that for me to read while I was sitting there, and then he explained it to me even more. And I decided right there and then, ‘Okay, fine. I will take it (PrEP)’, cause he (my PCP) was very open about it. You know, like I said, if it came different, I probably would’ve felt uncomfortable.*”

(women#20, 51 years)

On the other hand, some providers still hold biased perspectives regarding individuals with specific characteristics who will benefit from PrEP use. For instance, a provider said, *“I’m not seeing as many younger patients. They (younger patients) benefit more from PrEP than others. I think, for my own perspective is for patients who are at higher risk for HIV, either because, uh, they, uh, because their lifestyle, uh, because they engage in sex, uh-uh, sex, or, uh, because they’ve had a history of STDs, uh, would be the ideal people to address PrEP with.”* (PCP#17, 40 years, male, family medicine).

On the contrary, other providers tried to eliminate prejudice when screening patients for PrEP indications in their practice as a PCP said: *“I think it’s very important for healthcare providers to always provide that information to all their patients, regardless of their so-called situations. people should never have your own bias or automatically assume if someone tells you something that’s the whole story. Cause not everyone fits in the same picture. But the most important thing is that they know that that option’s available.”* (PCP#28, 36 years, female, advanced nurse).

Effective communication strategies with respect can successfully facilitate a smooth decision-making process regarding PrEP use in the primary care settings, as a provider described: *“I don’t like just to spit out information. I like to be interactive and do the teach back, so I can, so I can fully, um, assess that they understand what’s goin’ on and what the, the medication’s about, how it works… I always have a pamphlet, um, showing the difference between them and the pill sizes…. When they can see the numbers, see the graph, it can help—it helps them understand a little bit better than me just verbally explaining.”* (PCP# 28, 36 years, female, advanced nurse). 

Another provider echoed this point using another example: “*I confirm that they (the patients) are here for PrEP, but then I don’t go there right away. I just treat it as a regular visit to kind of just establish some, uh, rapport. Not to just immediately jump into, like, sexual health. Going through the appropriate steps, um, just getting a medical history first. Just check everything, hepatitis panel, gonorrhea, chlamydia, syphilis. we squeeze them in to be seen. Um, or I get, like, a prescription request, uh, request from the pharmacy*.” (PCP#30, 36 years, female, infectious disease specialist).

Trust in providers and the health system facilitates emotional bonds and establishes collaborations between providers and patients, which is further enhanced by lines of communication [54]. A PCP emphasized the impact of trust in PrEP care and described how she fostered a trustworthy relationship with her patients when prescribing PrEP in the primary care settings: *“I love prescribing PrEP. Because I find that, um, for one thing patients really trust you when you’re willing to kind of, um, meet them at that level, and have those kinds of conversations with them. I’ve always found that I develop much stronger, patient/clinician relationships with patients when I’m doing, um, PrEP. Um, a lot of times it-it might be patients who have previously had poor experiences in the healthcare system, or younger patients that just really haven’t had much of any experience in the healthcare system. Um, so it’s really nice to be able to develop those relationships with, uh, folks.”* (PCP#14, 38 years, female, family medicine).

Similarly, patients also reported their experience about how the therapeutic alliance can be formed via non-discriminated and non-judgmental communications: *“it was her (who initiated the discussion about PrEP). It was her that started the conversation due to the fact that I was from another clinic and she saw on my, uh, on my files that. And she asked me if I knew about PrEP. I didn’t feel, um, discriminated. (So in other, uh, occasions, uh, other doctors I had, they would, uh, uh, tell me, like, uh, you know, like, why don’t—why, you know, like, they made me feel that it was, um, they made me feel like, uh, it was wrong for me to be with someone that was HIV positive) she encouraged you for you to be safe.”* (women#11, 52 years)

#### 3.2.2. Sexual Wellness and Sexual Health Exploration in the Primary Care Settings 

Sexual history exploration is crucial for determining patients’ eligibility for PrEP use. However, conversations about sexual wellness and sexual history posed a challenge in healthcare settings reported by providers and patients. In our study, about one-third of providers expressed various concerns (e.g., discomfort, embarrassment) about the discussion about the sexual history or sexual wellness. In particular, one PCP explained: *“I don’t see it (PrEP) ever on anyone’s, um, medication list. Um, you know, personally how I would practice, and it would be—I would have to just kinda figure out how to bring it up in conversation about addressing that issue, um, of needing PrEP. I don’t really talk about people’s sexual history unless they have a sexual health complaint. I can’t speak for the physicians that—in my practice, um, you know, but I do find that the sexual history is often blank when reviewing a chart.”* (PCP#2, 33 years, female, primary care). 

Although some providers sought information about patients’ sexual activities, the exploration was not comprehensive enough to estimate patients’ actual risks for HIV infection: *“I do talk about sexuality. I don’t always get into every specific sexual practice that a patient may or may not do, and I-I think for me it’s on a need to know basis. I never wanna be in a space of being voyeuristic with one of my patients. I mean probably blind spots are a barrier. You know, assumptions of, oh, this person is 55. They’re not at risk. not even going-not even going to the questions about relationships and sexuality.”* (PCP#16, 54 years, female, primary care). 

In addition, competing clinical priorities served as another barrier to sexual history exploration. About one-third of providers reported either time or competing priorities hindered their PrEP care practice, as a PCP explained, *“you know, most of us probably don’t take a sexual history at every visit or do screening for risky sexual behavior, um, as often as we could. There’s a thousand other things to do in a visit when you’re seeing someone and they’re coming in with their, uh, laundry list of problems.”* (PCP#23, 46 years, male, primary care).

On the other hand, very few patients had discussed sexual history with their providers, and they expected their providers would initiate the sexual wellness-related topics. For instance, a patient mentioned, *“Neither has my primary care, neither, nor my OB-GYN has really never asked me about my relationship, my sexual history. I would imagine that might have something to do with the reason I’ve never heard of it (PrEP)… I guess I never, uh—you know, you don’t think about it (PrEP) until someone else brings it up to you about why they wouldn’t have done that.”* (women#18, 33 years). 

In the current study, we identified a “discussion paradox” in PCP, and patients considered that initiation of the topics regarding sexual wellness and PrEP eligibility screening should be the other side’s responsibility. This theme echoed how communication functions (e.g., information exchange and managing uncertainties) led to the proximal and intermediate outcomes regarding PrEP use in primary care settings [47,48,49]. A PCP explained why they were reluctant to explore the sexual history and discuss PrEP with patients: *“I think we rely heavily that those that are, um, that-that women especially are seen by a GYN and that they will kind of take care of that. And it’s not something that we’ll necessarily need to deal with. I don’t know if it’s just uncomfortable to bring up or I know that a lot of patients that are higher risk for HIV or have partners with HIV go to the HIV clinic at the <School>. And that’s probably where a lot of physicians would refer them because they just might not be as comfortable prescribing PrEP.”* (PCP#2; 33 years, female, primary care). 

Another patient assumed several reasons (e.g., unawareness, fear of stigma, discomfort) that their providers had not brought up PrEP use-related topics: *“I would assume that doctors would bring it up and, like, inform their patients I just—I never heard of it, so I never brought it up, and the doctor also didn’t bring it up. maybe they(PCP) don’t know about it. Maybe they—maybe it’s not part of their standard practice to bring up unless the patient brings it up. maybe they don’t want to, like, offend patients by insinuating maybe that they’re promiscuous or, like, something like that. Those are the reasons I can think of why they wouldn’t bring it up. So I think definitely, like, feeling comfortable with the specific doctor and the specific practice would make me more likely to bring it (PrEP use) up.”* (women#31, 20 years).

Although the “paradox” has been consistently reported by providers and patients in the current study, some providers exerted great examples of how efficient communications could resolve the block smoothly.

“*I had a new patient visit, um, a couple of days ago that, um—it was actually kinda easy because I asked—um, the patient had mentioned that he had a perianal strep infection. And so, um, you know, that was awkward for him, and-and it was a new patient visit, so I think that made it a little bit more awkward for him. And, um, that just kinda segued us into the discussion about sexual health, and I asked him, you know, “Have you heard of PrEP?” And he had been on it on the past. Um, asked him, you know, was he interested in restarting in it because he doesn’t have—he didn’t have a PCP. He was establishing PCP with me, and so he was-he was interested in restarting it., kind of an easy, um, discussion.*”

(PCP#15, 28 years, male, family medicine)

On the other hand, the majority of interviewed patients indicated that they would be happy to discuss PrEP-related topics with their providers if the providers could bring up this topic; one of them explained:

“*I never heard of it (PrEP) except for in ads about HIV. And I thought it was more for people who have HIV or for people who were very greatly at risk. So I didn’t really put myself into those categories. And, um, I was—but, yeah. I feel like I have heard of PrEP, like on T.V. I don’t feel uncomfortable or anything. I would do something like that if it was something I felt was beneficial for me. I mean, I guess it would just be them either bringing it up or bringing up, like—like, if they said, like, ‘Have you ever heard of PrEP?’ Or like if they say something like, ‘It seems you’re at risk for HIV.’ at that point, I would probably go, ‘Is there anything I can do about it?’ Or “What is PrEP?” Like, I guess those would be the two things that would make me to think about it.*”

(women#32, 21 years)

## 4. Discussion

The current study is among pioneering research [30,37,55] to qualitatively explore how specific communication pathways and interpersonal relationships between health providers and their patients impacted the PrEP care implementation in the primary care settings using the “Communication Pathway” Framework. Our analyses showed that communication functions affect health through proximate outcomes (e.g., trust in providers, patients’ satisfaction) that could affect intermediate outcomes (e.g., linkage to care, shared decision making) that lead to desired health outcomes (e.g., improved sexual wellness and reduced HIV risks) or healthcare utilization (e.g., PrEP uptake) [56,57,58].

The interpersonal relationship and mutual trust between providers and patients enhance patients’ satisfaction and collaboration in health care settings to achieve sustainable care and optimal health outcomes [59,60]. Furthermore, clinical decisions may be impacted by the complexity of evidence and the perceived risks and benefits regarding the chosen treatment regimens from both patients and providers [59,60]. Communication about health risks requires an appropriate conveying format to help the audience understand their risk magnitudes and how they are likely to be impacted and prevented [61]. Patients’ risk perceptions are synergistically combined with their own experience, scientific evidence obtained from various sources (e.g., social media, clinical consultations), and conscious and unconscious cognition [62]. On the other hand, health providers’ perceptions may be interpreted by clinical evidence through the lens of their personal experience and their biased and unbiased heuristics [59,61]. Communication serves as a gateway to exchange information, understand each other’s values and needs, reconcile disagreements, and finally achieve a mutually acceptable and feasible treatment plan in the pathway toward better health outcomes [33]. 

In the current study, our data showed that patients usually considered themselves to have relatively low risks of HIV infection despite consuming high-risk behaviors. They generally had suboptimal PrEP awareness and misconceptions about PrEP candidacy (e.g., “only gay men and promiscuous persons need PrEP”). PCP reported time constraints and competing priorities (e.g., other more urgent health issues) in clinical care compared with PrEP consultation and PrEP care. They also tended to make ungrounded assumptions about their patients’ risk profiles (e.g., “unnecessary to explore”; “their risk is low”) and adverse reactions (e.g., refusal, embarrassment) from their patients about the PrEP discussions at the primary care settings. These barriers may block the pathways via proximal (e.g., mistrust) and intermediate outcomes (e.g., linkage to care) from communication functions to health utilization outcomes. On the other hand, our data revealed that effective and strategic communications (e.g., pamphlets, patient-centered approach) could remove these blocks along the pathways. Our findings resonate with other researchers’ work [63,64,65]. For instance, Brown et al.’s (2016) review indicated that trust was a hard-to-capture but critical predictor for optimal treatment outcomes (i.e., patient satisfaction, acceptance, and adherence to medical interventions or treatments) [64,65]. Therefore, we should make efforts to enhance the communication functions in clinical settings, especially in primary care settings, where providers encounter HIV-negative patients more often than in HIV and STD clinics [29]. 

As other researchers indicated, discussions regarding sexual history and PrEP eligibility posed a challenge in primary care settings [37,66]. Our data suggested that the procedure of sexual wellness exploration was usually superficial, and no in-depth conversations could be initiated and proceeded. Both patients and PCP were reluctant to discuss these sensitive topics as they assumed the other party may feel discomfort. Meanwhile, they expect the other side can bring up the sexual wellness topics. On the other hand, patients and providers expressed the willingness to discuss these sensitive topics as long as the other side could initiate the conversation. In the primary care settings, the “discussion paradox” may cause missed critical opportunities to evaluate individuals at risk of HIV infection and use PrEP as prevention, especially for groups with the highest risk of HIV acquisition [67]. As a result, a screening tool for PrEP candidacy could serve as the bridge to facilitate the initiation of the conversation. We call for efforts to develop an easy-to-use and reliable tool via appropriate channels to collect PrEP eligibility information and pave the way for the discussion in the primary care settings [58]. In addition, PrEP care navigators could share logistic burdens for providers and facilitate the care cascade smoothly in the clinical settings [37]. 

There are several strengths in the current study. First of all, we analyzed the data based upon the “Communication Pathway” Framework. Unlike most available research focused on facilitators and barriers along the PrEP care cascade [30,37], we explored how patient–provider communication impacts health outcomes and health utilization via specific pathways. Second, we analyzed points of view regarding the PrEP care cascade from both providers and patients. Unlike studies that only collected data from one side [30,37,55], we can describe the overall picture holistically using data collected from all stakeholders. Third, most research examined PrEP care in clinical settings in general [1,49,63]. Only a few, including this current study, examined the specific role of PCP in the PrEP care cascade [65], who play as a gatekeeper to identify PrEP-eligible patients in routine care. Fourth, we employed both thematic and content analysis approaches to achieve more rigorous findings than using either method alone [45]. Using the content analysis, we explored the multifaceted phenomena of communication pathways and identified the quantitative patterns [68]. As a flexible and valuable approach, we employed thematic analysis to describe the detailed and nuanced life stories of both patients and PCP [69]. 

Several caveats in the current study have been acknowledged, and we must interpret the findings cautiously. First, we employed a convenience sample strategy to recruit PCP who practice in NYS and women who reside in NYS. The sampling scheme may be subject to selection biases, as only individuals who were interested in PrEP would participate. However, individuals unfamiliar with PrEP or HIV prevention strategies may bear higher risks of HIV infection than those recruited in the current study. Therefore, results from the current study may not be generalizable to providers and patients living in other States, especially in States that share top-heavy burdens in HIV epidemics [70]. We called for a nationwide study using the randomized sampling strategy to understand better the pattern of PrEP implementation in primary care settings. Second, due to the limited scope of the current study, we did not explore specific pathways among individuals with diverse race/ethnic and other key characteristics. Research indicated that individuals of different races/ethnicities reported various levels of barriers along the pathways [71]. We call for larger-scale research in the future to explore the specific pathways among individuals with various background characteristics. Third, although we try to frame data to align with the socioecological model, we did not collect clinical- and community-level data to explore how these factors impact the specific pathways in the PrEP implementation cascade. Fourth, despite the fact that we had reached sufficient information power with the recruited sample size in the current study, we call for a study with a broader scope and larger sample size to provide a more comprehensive picture of PrEP care implementation among PrEP-eligible individuals. 

## 5. Conclusions

Our findings indicate that both health providers and patients need skills and assistance to express their points of view while incorporating others’ perspectives to increase knowledge and linkage to essential care and establish a reliable patient–provider alliance with shared understanding. A PrEP care navigation and assistance system or a PrEP screening tool may fill the gaps regarding PrEP eligibility screening and consultation facilitation in primary care settings. The future effect would benefit from facilitating PrEP discussion, engagement, and monitoring for all stakeholders in primary care settings. 

## Figures and Tables

**Table 1:** Demographics and key characteristics of included participants.

Primary Care Providers (*n* = 18)		PrEP-Eligible Women (*n* = 29)	
Age (mean, * SD; range)	42.4 (SD = 12.9; 28–68)	Age (mean, * SD; range)	38.1 (SD = 15.1; 20–61)
Year of Practice (mean, * SD; range)	9.0 (SD = 10.9; 1–40)		
Gender (*n*, %)		Number of sex partnership (mean, * SD; range)	1.6 (SD = 1.1; 0–5)
*Male*	4 (30.7%)		
*Female*	13 (72.2%)	Sex without condoms (*n*, %)	23 (79.3%)
*Others*	1 (5.6%)		
Race/Ethnicity (*n*, %)		Living with HIV positive partners (*n*, %)	6 (20.7%)
White	17 (94.4%)		
Black	1 (5.6%)	Reported sexually transmitted infections in the past 6 months (*n*, %)	2 (6.9%)
Practice Places (*n*, %)			
*Hospital-based*	13 (72.2%)	Ever used substances (e.g., alcohol, cocaine, or other drugs) (*n*, %)	5 (17.2%)
*Federally qualified health center*	3 (16.7%)		
*Group practice*	3 (16.7%)	Ever injected drugs (*n*, %)	7 (24.1%)
*Kaiser Permanente*	2 (11.1%)		
Primary Specialty (*n*, %)		Ever had HIV testing (*n*, %)	20 (69.0%)
*Family Medicine*	10 (55.6%)		
*Primary care*	7 (38.9%)	Ever used PrEP (*n*, %)	3 (10.3%)
*Internal Medicine*	3 (16.7%)		
*Infectious Disease*	2 (11.1%)		
*Adolescent Health*	1 (5.6%)		

* SD: Standard Deviation.

**Table 2:** Key findings based upon the conceptual framework.

Thematic Domains	Specific Pathways
**3.2.1 Provider–patient communication and PrEP care implementation**	Via Patient knowledge (i.e., informational exchange)
	Via Therapeutic alliance (i.e., proximal outcomes)
	Via Linkage to care (i.e., intermediate outcomes)
	Via Decision making on PrEP care (PrEP implementation)
**3.2.2 Discussion paradox**	Initiation of the PrEP care
	Sexual wellness exploration

## Data Availability

The data presented in this study are available on request from the corresponding authors. The data are not publicly available due to privacy concerns and the protection of sensitive information from the research subjects.
